# Bisphenols and Risk of Breast Cancer: A Narrative Review of the Impact of Diet and Bioactive Food Components

**DOI:** 10.3389/fnut.2020.581388

**Published:** 2020-11-19

**Authors:** Barbara J. Stillwater, Ashleigh C. Bull, Donato F. Romagnolo, Leigh A. Neumayer, Micah G. Donovan, Ornella I. Selmin

**Affiliations:** ^1^Department of Surgery, Breast Surgical Oncology, University of Arizona, Tucson, AZ, United States; ^2^School of Medicine, University of Utah, Salt Lake City, UT, United States; ^3^Department of Nutritional Sciences, University of Arizona, Tucson, AZ, United States; ^4^University of Arizona Cancer Center, Tucson, AZ, United States; ^5^Department of Surgery, University of Florida College of Medicine-Jacksonville, Jacksonville, FL, United States

**Keywords:** breast cancer, epigenetics, nutrition, bisphenol, estrogen receptor (ER)

## Abstract

Data from preclinical studies suggest a link between increased risk of breast cancer and exposure to bisphenols at doses below what the United States Food and Drug Administration (FDA) considers as safe for consumption. Bisphenols exert estrogenic effects and are found in canned and plastic wrapped foods, food packaging, and plasticware. Mechanistically, bisphenols bind to the estrogen receptor (ER) and activate the expression of genes associated with cell proliferation and breast cancer. In this paper, we present a narrative literature review addressing bisphenol A and chemical analogs including bisphenol AF, bisphenol F, and bisphenol S selected as prototype xenoestrogens; then, we discuss biological mechanisms of action of these bisphenols in breast cells and potential impact of exposure at different stages of development (i.e., perinatal, peripubertal, and adult). Finally, we summarize studies detailing interactions, both preventative and promoting, of bisphenols with food components on breast cancer risk. We conclude the review with a discussion of current controversies in interpretation of the above research and future areas for investigation, including the impact of bisphenols and food components on breast tumor risk.

## Introduction

**Figure 1:**
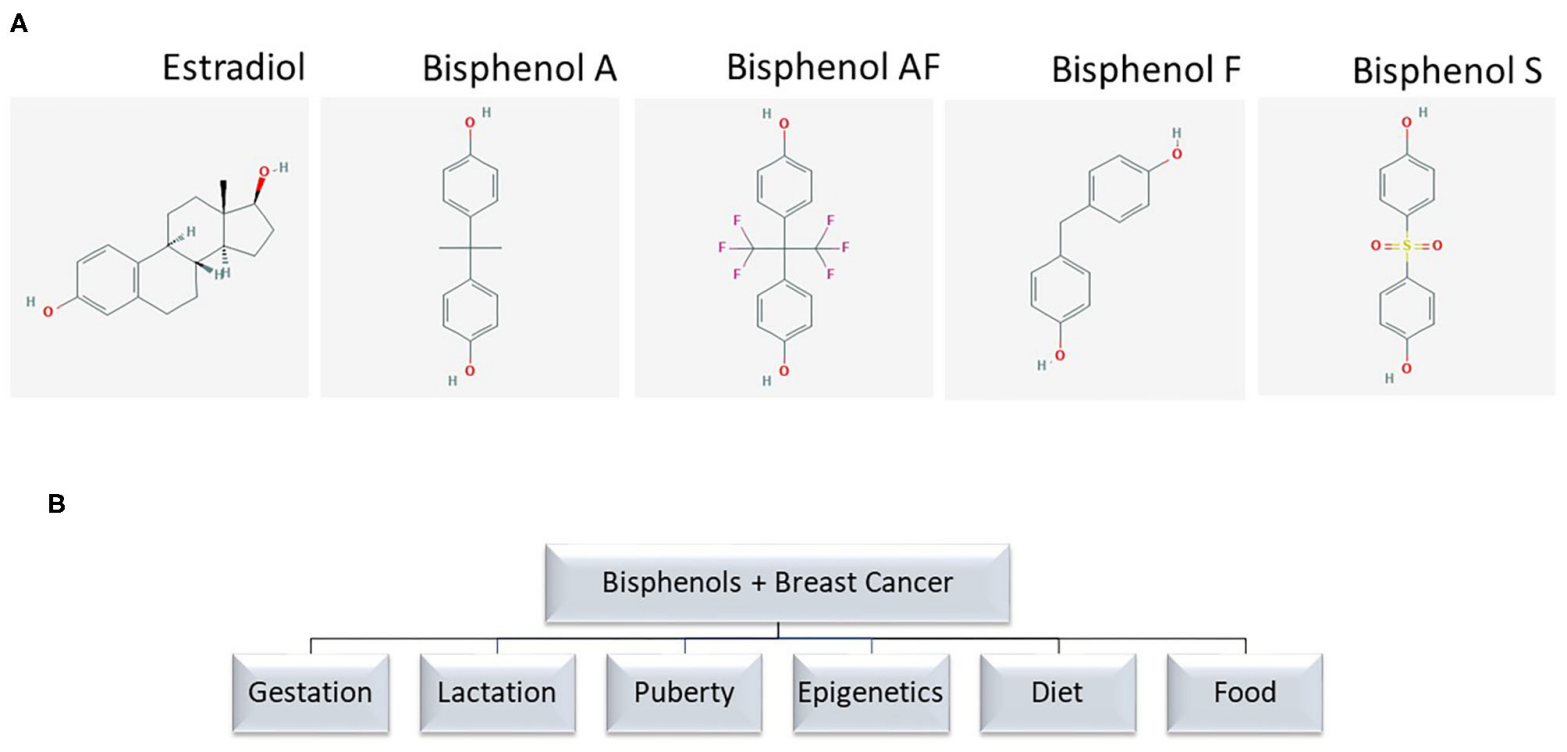
**(A)** Chemical structure of bisphenol analogs. Chemical structures are from PubChem. **(B)** Terms used for PubMed search.

Through the environment and foods, women are exposed to xenobiotics, which can exert exogenous endocrine effects on the body. These endocrine disruptors that exert estrogen (E2)-like effects may influence breast cancer risk, and include polychlorinated biphenyls, pesticides, and plastic additives. One such plastic additive is bisphenol A (BPA), which shares chemical similarities with other bisphenols such as bisphenol AF (BPAF), bisphenol F (BPF), and bisphenol-S (BPS) (Figure 1A). Bisphenol A (BPA) is an industrial chemical primarily used in the production of polycarbonate plastics and epoxy resins (1). Polycarbonate plastics are used in water bottles, toys, CDs, food and beverage packaging, plastic tableware, flame retardants, medical, dental, optical devices, computers, wire insulation, and thermal paper (2–4). Epoxy resins are used as lacquers to coat metal products like food cans, bottle tops, and water supply pipes (2, 3). BPA is also found in dust, laminate flooring, paints, and home electronics (2). Classified as endocrine disrupting chemicals (EDC) due to their structural similarity to E2, bisphenols can interfere with normal endocrine functions depending on dose and timing of exposure throughout life. Studies have examined the biological effects of exposure to bisphenols during various stages of life such as gestation, lactation, and puberty, and as causative agents in the development of endocrine tumors, especially of the breast. Breast cancer is the most common cancer in women aside from skin cancers, and 12% of women in the United States will receive this diagnosis in their lifetime. Breast cancer has three major clinical subtypes that matter to clinicians, patients and families alike: 70% of breast cancers are hormone (estrogen and progesterone) receptor positive, 15–20% are human epidermal growth factor (ERBB2) positive (formerly HER2), and 15% are “triple negative” or do not possess receptors for homones or ERBB2 (5). These clinical subtypes determine treatment and prognosis in most cases of breast cancer. BPA, as an EDC, has been studied as a causal agent in these cancer subtypes with varying effects depending on the dosage and clinical subtype.

The objectives of this review are (1) to summarize recent original research evidence form preclinical and clinical studies related to biological mechanisms of bisphenol interference with regulation of endocrine functions in the breast; and (2) discuss biological interactions of food components with bisphenols and their impact on breast cancer risk.

## Methodology

We searched PubMed to identify relevant studies published in English on the relationships between BPA and BPS exposure and breast cancer cross-checked with references for selected search terms (Figure 1B). Reviews and original research published before 2010 are excluded here except to direct the reader to two significant studies related to mechanisms of action or human exposure to bisphenols linked to breast cancer.

## Results

### Sources of Exposure

Food packaging represents the major source of human exposure to bisphenols. A list of foods containing bisphenols and relative concentrations is provided in Table 1 (6–9). The hydrolysis of the ester bonds that link bisphenol monomers is accelerated by heat, as well as acids and bases (2). As the monomers are freed, they are leached from the plastic packaging into products, especially food and water. Because hydrolysis occurs with processes essential to food preparation, transport, and storage, bisphenols are found ubiquitously in human diet.

**Table 1:** Bisphenol concentrations found in foods.

**Foods**	Mean or range^**^	**Mean**
	**BPA level**	**BPS level**
	**ng/gm**	**ng/gm**
**Canned Foods**		
Refried beans^*^	6.3–790	^***^
Green beans^*^	22–730	^***^
Green beans^*^	18.0	ND
Green peas^*^	3.1–310	^***^
Green peas^*^	30.0	ND
Del monte fresh cut green beans	26.6–65.0	^***^
Creamed soup^*^	41.0	ND
Portuguese canned mackerel	36.3	^***^
Baked beans^*^	36.0	ND
Progresso light homestyle vegetable and rice soup	15.6–22.7	^***^
Meat broth^*^	23.0	ND
Portuguese canned tuna	17.7	^***^
Progresso classics vegetable soup	7.3–11.7	^***^
Evaporated milk^*^	11.0	ND
Progresso classics tomato basil soup	8.2–10.7	^***^
Campbell's condensed chicken noodle soup	4.5–7.1	^***^
Hormel chili with beans	3.5–5.6	^***^
Kroger sweet peas	2.7–4.0	^***^
Chicken of the sea chunk light tuna in water	1.7–3.8	^***^
Kroger mixed vegetables	2.3–4.2	^***^
Campbell's chunky savory pot roast	1.5–2.0	^***^
Kroger canned beef	0.8–1.7	^***^
Enfamil premium LIPIL infant formula milk based	1.0–1.2	^***^
Beach cliff sardines in water	0.8–1.3	^***^
V8 100% vegetable juice	0.7–0.8	^***^
Hormel spam	<0.2–0.3	^***^
Similac advanced infant formula	<0.2	^***^
Chef boyardee mac and cheese	<0.2	^***^
Bumble bee chunk light tuna in water	<0.2	^***^
		^***^
**Plastic Wrapped Foods**		^***^
Chef boyardee spaghetti and meatballs	4.3–5.0	^***^
Sliced turkey^*^	0.35	^***^
Sliced chicken breast^*^	<0.2	^***^
Sprouts organic cinnamon applesauce	<0.2	^***^
**Unpackaged and Non-Canned Foods**		
Ground beef^*^	ND	35.0
Beef steak^*^	ND	18.0
Organ meats^*^	ND	7.6
Roast beef^*^	ND	7.1
Veal cutlets^*^	ND	6.9
Pork^*^	ND	5.1
Sausages^*^	ND	3.3
Cold cuts^*^	ND	2.7

BPA and BPS levels in various foods (5–8).

* Brands not identified.

** The range of identified brands represents the highest and lowest levels of BPA from samples from three cans. The range for foods in unidentified brands represents the lowest and highest levels of BPA from two to 10 different brands of the same food.

*** Not measured.

*ND, Not detected*.

Canned foods are a significant source of dietary exposure. Exposure to BPA from daily use of plastic water bottles is ~0.17 μg/d, and from food cans ~20 μg/can (10). About 70% of canned fruit and vegetables samples have a BPA content in the range of 0.8 ng/g for V8 juice to up to 790 ng/g (canned refried beans), with the second and third highest levels as high as 730 ng/g in canned green beans and as high as 310 ng/g for canned green peas. Mean BPA levels in canned fish average 7–12 ng/g in the US (6, 11); 33 ng/g in Europe (12); and 106 ng/g in Canada (13). The estimated total adult exposure to BPA from food ranges from 30 to 70 ng/kg/d (14).

While metal does not typically contain bisphenols, food cans are lined with epoxy resins, which contain bisphenols (e.g., BPA) in order to provide a malleable structure to the plastic lining. Monomers of BPA are known to leach into contained food through heat used for sterilization or food preparation (2, 15). Similar to epoxy resins, leaching from polycarbonate plastics is increased if the container is heated. If polycarbonate plastic is used in packaging such as water bottles, food cans, infant bottles, or formula cans, heating is likely to increase human bisphenol exposure from the food.

The relationship between the type of packaging and the amount of detectable BPA and/or BPS is demonstrated in the table (Table 1). The amount of BPA in canned and plastic wrapped foods varies widely by manufacturer and their canning processes. In this table, the presence of BPS appears to be limited to unpackaged foods.

#### Governmental Regulation of BPA

Since 2012, the FDA has banned BPA from use in plastic baby bottles, sippy cups, and baby formula packaging (1). However, based on scientific review, the FDA, as of 2018, has “not found any information to prompt a revision of FDA's safety assessment of BPA in food packaging” (16). Therefore, its use continues in non-infant applications such as food packaging.

BPA is classified by the US Environmental Protection Agency as an endocrine disrupting chemical (EDC). The US Environmental Protection Agency (USEPA) has set an oral Reference Dose (RfD) for BPA at 50 μg/kg bw/day (17). The RfD is defined as an estimate of daily oral exposure that is likely to be without significant risk of negative lifetime effects. However, this recommendation only takes into account daily oral intake, and does not address potential exposure *in utero* or as an infant, exposure from environmental hazards, or accumulated stores in adipose tissue that are gradually released over time (18). The USEPA plans to gather data with respect to environmental effects of BPA to further determine whether “BPA either does or does not present an unreasonable risk of injury to the environment” (19). In 2016, the European Chemicals agency identified BPA as a substance of very high concern regarding its use in thermal paper and thus BPA was added to REACH Annex XVII Restricted Substances List. This new entry bans BPA's use in thermal paper with a concentration equal to or above 0.02% by weight (20).

### Mechanisms of Action

BPA was first synthesized in 1891 by Russian chemist Alexander Dianin. However, its estrogenic properties were not recognized until 1932 by British chemist, Charles Dodds. In 1934, Dodds compounded a more stable form of synthetic estrogen, showing that a synthetic estrogen could mimic natural estrogen in animals. He later went on to synthesize diethylstilbestrol or DES, an artificial estrogen that is a structurally similar to BPA. In 1971, DES was banned for use during pregnancy in the US due to increased risk of endometrial and other cancers in female fetuses. However, DES was still used as a contraceptive and as a treatment for post-partum lactation suppression. Although studies of DES in the 1970's linked DES exposure to cancers of the female lower genital tract, it was not until 1985 that the FDA listed DES as a known carcinogen (21).

Excellent reviews of biochemical mechanisms of actions of BPA are available elsewhere (22). Due to structural similarity to estradiol (Figure 1A), bisphenols act through a *genomic mode* of action via binding to the estrogen receptors ERα and ERβ (23), though bisphenols have lower affinity for these receptors than naturally synthesized human estrogens. For example, compared to the activity induced by 1 nM 17-β-estradiol (E2), a 50% activation of ERα requires a concentration of BPA approaching ~1.3 μM, raising the question as to whether or not micromolar concentrations of BPA and other bisphenols are achievable in humans through chronic or acute exposure (24).

Studies have shown that in the MCF-7 breast cancer cell line, BPA binds with similar affinities to ERα (IC50 ~6.0 × 10−6M) and ERβ (IC50 ~6.5 × 10−6M); elevates transcriptional activity at estrogen response elements; and exerts proliferative effects (25). The MCF-7 cell line has been shown to express both ERα and ERβ (26). Although ERβ has been suggested as a cancer therapeutic target (27), some studies (28) show that when ERα/β positive MCF-7 cells are treated with an active metabolite of BPA [4-methyl-2,4-bis(4-hydroxyphenyl)pent-1-ene (MBP)] at levels (~1 nM) comparable to human environmental exposure, ERα protein expression is downregulated and proliferation is increased *via* ERβ. In particular, the MCF-7 proliferation stimulated by MBP is dose-dependently counteracted by the cotreatment with the selective ERβ antagonist PHTPP (4-[2–phenyl-5,7–bis (trifluoromethyl) pyrazolo [1,5-a]-pyrimidin-3-yl] phenol). Additional support for a positive role of ERs in the BPA-induced proliferation is offered by another study showing that in MCF-7 cells, BPA transactivates both ERα and ERβ (29). This cumulative evidence suggests that parent bisphenol compounds and metabolites may exert proliferative effects on breast cells at physiologically relevant (<1.0 nM) concentrations.

In addition to binding to nuclear ER, bisphenols activate signaling pathways through *non-genomic mechanisms*. In ERα-positive (30) and triple-negative breast cancer (TNBC) cells (MDA-MB-231) (31), BPA activates signal transduction pathways [i.e., extracellular signal-regulated kinase 1/2 (ERK1/2)] involved in proliferation *via* G-protein coupled estrogen receptor (GPER) and epidermal growth factor receptor (EGFR). Using pharmacological inhibition and gene-silencing approaches, studies by Pupo et al. (32) document that BPA induces in SkBr-3 breast cancer cells the expression of the GPER-target genes c-FOS, early-growth response protein 1 (EGR-1), and connective tissue growth factor (CTGF) through the GPER/EGFR/ERK signal transduction pathway. Via GPER, BPA induces cell proliferation and migration of TNBC cells (SkBr-3 and MDA-MB-231) and enhances tumor growth *in vivo* (31). In hypoxic conditions, BPA promotes hypoxia inducible factor-1-alpha (HIF-1α) and VEGF expressions through a GPER/Caveolin-1/heat shock protein 90 axis (33). Interestingly, ERα is activated and degraded by hypoxia in breast cancer cells. Conversely, BPA enhances ERα-mediated transcriptional activity under conditions of hypoxia. Since hypoxia is known to favor tumor progression, exposure to BPA and other bisphenols exacerbates resistance to endocrine therapy via GPER-dependent mechanisms (24). Low levels of BPA (≤ 10^−8^ M) induce phosphorylation of protein kinase D1 (PKD1), which is associated with increased activation state of PKD1 (34). These observations suggest that exposure to bisphenols at physiologically relevant concentrations induce the growth of ER-positive and ER-negative breast cancer cells through genomic and non-genomic mechanisms. The following sections summarize research evidence related to effects of bisphenols on endpoints of breast cancer from preclinical and clinical studies. While most studies have focused on BPA, when available comparative data about its analogs BPAF, BPF, and BPS are included.

### Cell Culture

#### BPA

In ERα-positive breast cancer cells (MCF-7, T47D), BPA increases expression of p53 and ERα in a concentration-dependent manner. It also promotes cell proliferation which is hampered by the ERα-antagonists tamoxifen and ICI (35). BPA supports the growth of ERα-positive tumors by inducing heat shock factor-1 (HSF1) phosphorylation on S326 *via* mitogen-activated protein kinase (MAPK)/ERK1/2 (MEK1/2) signaling (36). BPA, along with other endocrine-disrupting compounds, increases aromatase expression and activity leading to increased levels of 17β-estradiol and proliferation in ERα-positive breast cells (37). BPA and 4-cumylphenol (4-CP), another E-like compound, at levels ranging from 10^−9^ to 10^−5^ M, stimulate cell proliferation and expression of ERα, pS2, and B-cell lymphoma-2 (Bcl-2) in MCF-7 breast cancer cells. In addition to exerting E2-like effects, studies with ER-positive breast cancer cells show BPA antagonizes the proapoptotic effects of tamoxifen while favoring transition of cell cycle from G1 to S phase, and upregulating cyclin D1 (CCND1) and ERα. Expression of estrogen related receptor y (ERRγ) and its coactivators peroxisome proliferation-activated receptor γ coactivator-1α (PGC-1α) and PGC-1β are also increased. In turn, binding of ERR-y to BPA protects the latter from deactivation *via* 4-hydoxytamoxifen (2).

These data support the notion that exposure to BPA and possibly other bisphenols increases proliferation while hampering the efficacy of endocrine therapies (38). In MCF-7 cells exposed to nanomolar concentrations (10 nM) of BPA there is accumulation of aldehyde dehydrogenase 1 (ALDH1), a marker of human mammary stem cells, and increased growth of mammospheres. These effects are not observed in ER-negative (MDA-MB-231) breast cancer cells. Mechanistically, BPA induces expression of SRY-related HMG box-containing transcription factor-2 (SOX2), a factor that promotes cell proliferation and metastasis. These findings denote that physiologically relevant levels of BPA increase growth of ER-positive breast cancer through stem-like cell activity via upregulation of SOX2, which participates among other factors [e.g., octamer-binding transcription factor-4 (OCT4)] in the maintenance of pluripotency of breast stem cells and an undifferentiated cellular state (39).

Endogenous estrogens and bisphenols from the environment exert overlapping effects. Results of association studies with E2 and BPA (~200 nM) indicate synergistic rather than additive activating effects on ERα, phosphatidylinositol-4,5-bisphosphate 3-kinase catalytic subunit alpha (PIK3CA), GPER, and phosphatase and tensin homolog (PTEN); and antagonizing effects on protein kinase B (Akt1) in breast cancer MCF-7 cells. Similarly, the co-exposure of lower concentrations BPA and 4-CP significantly induce cell proliferation in a synergistic fashion. Therefore, the combined effects and not simply individual exposures need to be considered to develop accurate models of breast cancer risk associated with exposure to bisphenols (40, 41).

Bisphenols exert biological effects based on breast cancer subtype. For example, subsequent to exposure to BPA (10^−8^ M), high expression of interleukin-19 (IL19), carbonic anhydrase-9 (CA9) and secreted protein acidic and rich in cysteine (SPARC) is seen in MCF-7, SK-BR3, and MDA-MB-231 cells, respectively. These gene expression signatures are believed to predict poor overall survival in luminal A, epidermal growth factor receptor-2 (HER2)-enriched and TNBC patients, respectively (42). The IL-19 protein promotes proliferation and metastasis in breast cancer cells (43). CA9 catalyzes the hydration of carbon dioxide to carbonic acid and is expressed mainly in high-grade, steroid receptor negative/HER2 enriched tumors. Its role in breast tumorigenesis is attributed to reducing pericellular pH in response to hypoxia, thus aiding in the degradation of extracellular matrix. These biological effects may contribute to lack of response to traditional therapy by breast tumors expressing high levels of CA9. Expression of SPARC is associated with invasion and is characteristic of more aggressive phenotypes (i.e., TNBC) (44). These expression results point to differential effects of BPA on processes associated with breast cancer including impaired immune response, and enhanced invasion and metastasis.

Inflammation is a process induced by BPA, which triggers cyclooxygenase-2 (COX-2) expression via nuclear translocation of nuclear factor kappa-light-chain-enhancer of activated B cells (NF-κB) and activation of MAPK/ERK1/2 in TNBC (MDA-MB-231) cells (45). Overexpression of EGFR/HER2 along with ER negativity is common in inflammatory breast tumors. BPA increases EGFR/ERK signaling, culminating with increased expression of superoxide dismutase 1 (SOD1) and anti-apoptotic Bcl-2, key markers of antioxidant and anti-apoptotic processes, respectively. BPA potentiates clonogenic growth and tumor spheroid formation which are pathological characteristics of inflammatory breast tumors. Furthermore, BPA antagonizes the effects of EGFR inhibitors (46).

Exposure to bisphenols impinges on the process of invasion and metastasis via induction of matrix metalloproteinases (MMP), which are enzymes involved in degradation of extracellular matrix. In TNBC cells, BPA induces ERRγ expression, whose knock-down markedly attenuates BPA-induced expression of MMP-2 and MMP-9. Inhibitors of ERK1/2 (PD98059) and Akt (LY294002) attenuate BPA-induced ERRγ expression and cell invasion (47). BPA promotes migration, invasion, and an increase in the number of focal contacts. Finally, BPA induces an increase in activator protein-1 (AP-1)- and NFκB-DNA binding activity (48). Nanomolar concentrations of BPA promote *in vitro* migration and induce epithelial to mesenchymal transition (EMT) of ER-negative breast cancer cells associated with downregulation of fork head box A1 (FOXA1), which is a determinant of response to endocrine therapy. Further, BPA (10^−8^ M) significantly increases the phosphorylation of ERK1/2, p38-MAPK, and Akt in TNBC cells. Overall, these observations point to BPA as a promoter of EMT in ER-negative breast tumors (49).

#### BPA Analogs

Although some BPA is being replaced in industrial production, there remain concerns about similar or even more potent estrogenic effects of bisphenol analogs (3). BPS, which is absorbed directly through the skin, is found in thermal receipts and currency bills from 21 countries (50). In reference to the relative estrogenic activity of BPA substitute, BPAF is the most potent bisphenol, followed by BPB > BPZ ~ BPA > BPF ~ BPAP > BPS. The addition of ICI 182,780 antagonizes the activation of ER by BPA analogs. Transcriptome alterations resulting from exposure to BPA substitutes indicated that BPA analogs act as ERα agonists in MCF-7 breast cancer cells. These results support the concern that BPA alternatives are not necessarily less estrogenic than BPA in human ERα-positive breast cancer cells. In fact, BPAF, BPB, and BPZ may be even more estrogenic than BPA (51). At concentrations ranging from 0.1 to 10 μM, BPA, BPS, and BPF stimulate proliferation of MCF-7 clonal variant (MCF-7 CV) cells in the order BPA = BPS > BPF. These bisphenols are equally effective at inducing the expression of CCND1 and CCNE1, and induce migration and expression of N-cadherin (N-Cad), while reducing levels of E-caherin (E-Cad). Conversely, these responses are antagonized by the cotreatment with the ER-antagonist ICI 182,780. Thus, not only BPA but also BPS and BPF effectively activate pathways associated with breast cancer *via* ER-dependent mechanisms raising concerns that substitution of BPA in food packaging with these bisphenol analogs may not limit breast cancer risk (52). The treatment in culture (24 h) with BPA, BPF, and BPS increases (2–3 folds) the expression and activity of telomerase in MCF-7 (ERα-positive) but not in MDA-MB-231 (ERα-negative) cells, and this increase is prevented by cotreatment with ERα antagonists. These results suggest that the effects of bisphenols in ER-positive breast carcinoma are mediated at least in part by telomerase, whose increased expression associates with breast cancer development and progression (53).

In MCF-7 and T47D ER+ breast cancer cells, BPAF promotes cell growth concurrently with induced ERα transcriptional activity and amphyregulin (AREG) (54). BPF at low concentration (10 nM) significantly enhances in MCF-7 cells the protein expression of ERα, GPER, c-Myc, and CCND1, as well as phosphorylation levels of Akt, ERK, and proliferation (55). BPAF at 0.001–1 μM and BPF at 0.01–1 μM increase cell viability, DNA damage and ROS in MCF-7 cells. These biological effects are attenuated by the ROS scavenger N-acetylcysteine (NAC), indicating that ROS play a key role in the observed biological effects of BPAF and BPF on MCF-7 cells (56). BPF promotes *in vitro* proliferation of ERα-positive breast cancer cells (T47D) in a dose-dependent manner, with EC_50_ ~120 nM. The C-X-C chemokine ligand 12 (CXCL12) is up-regulated through ERα in T47D cells treated with BPF (57). BPF functions as a stimulator of ERβ1 (and ERα) transiently expressed in MDA-MB-231 and SK-BR-3 breast cancer cells (EC_50_ values for ERβ: 6.87 and 2.58 nM, respectively, and EC_50_ values for ERα: 24.7 and 181 nM, respectively) (58).

In MCF-7 breast cancer cells, BPS (10 μM) promotes cellular responses commonly elicited by estrogens. These include accelerated G_1_ to S phase transition through the cell cycle; increased CCND1 expression and phospho-retinoblastoma (p-Rb) levels; release of E2F transcription factor; and increased expression of CCNE2 and CCNA2. The BPS-induced Rb phosphorylation and cell cycle progression is antagonized by the cotreatment with the ERα inhibitor ICI 182,780 and cyclin-dependent kinase-4/6 (CDK4/CDK6) inhibitor PD 0332991 (59). In non-tumorigenic breast cells, BPS induces upregulation of EGFR, and increases proliferation (60). BPA and BPS are equipotent in disrupting the organization of the acinar structures, despite BPS being less estrogenic compared to BPA. In combination, BPA and BPS augment the capacity for non-tumorigenic breast cells to invade the lumen. These data suggest BPA and its BPS substitute affect mammary development and contribute to breast cancer development (61). Bisphenol S promotes the migration of TNBC cells *in vitro* through activation of YAP, a key effector of the Hippo pathway, by inhibiting its phosphorylation, which promotes YAP nuclear accumulation and up-regulation of its downstream genes such as CTGF and ANKRD1 (62).

### Animal Models Including Xenografts

In a xenograft study of DCIS (ductal cancer *in situ*), Kim et al. documented that exposure to an environmentally human-relevant low dose of BPA (2.5 μg/l BPA for 70 days *via* drinking water) yields a 2-fold increase in the growth rate of the primary tumor along with increased lymph node metastasis (63). Overexpression of PKD1 increase the growth of BPA-exposed breast tumor xenografts *in vivo* (34). Similar to E2, BPA (37.5 mg pellet/60-days release) promotes established tumor growth of MCF-7 human breast cancer cells subcutaneously injected into flanks of ovariectomized NCR nu/nu female mice (64).

The exposure *in utero* [gestational day (GD)-9-GD21] to BPA decreases the expression of members of the chemokine CXC family (*Cxcl2, Cxcl4, Cxcl14*, and *Ccl20*), interleukin-1 (*Il1*) gene family (*Il1*β and *Il1rn*), interleukin-2 gene family (*Il7* receptor), and interferon gene family (interferon regulatory factor 9 (*Ilr9*), as well as immune response gene 1 (*Irg1*). These changes underscore a general impairment by BPA of anti-inflammatory factors. Additionally, BPA lowers *Esr1* (ERα) and augments *Esr2* (ERβ), whose expression is linked to increased risk of breast cancer (65). In MMTV-erbB2 transgenic mice, a model of HER2-positive breast cancer, *in utero* BPA exposure (500 ng/kg) daily between GD11-GD19 induces in offspring mammary tumorigenesis, earlier puberty onset, accumulation of terminal end buds (TEB), and prolonged estrus phase. Increased proliferative mammary morphogenesis associates with accumulation of ERα, *p*-ERα, CCND1, and c-myc, concurrent with activation of *erbB2*, EGFR, erbB-3, *Erk1/2*, and Akt. In OVX female rat offspring, the gestational exposure of BPA (50 μg BPA/kg/day from GD9 until weaning) elicits a higher incidence of ductal and atypical lobular hyperplasia in combination with E2 replacement compared to E2 alone. Thus, the perinatal exposure to BPA increases the susceptibility of mammary tissue to developing hyperplastic lesions (66).

The perinatal exposure to BPA increases the risk of proliferative lesions in offspring. In Wistar rats, *in utero* BPA exposure starting at GD7 through GD21 followed by lactational exposure through PND 22, induces mammary outgrowth in males at a low dose (0.025 mg BPA/kg body weight/d). Increased prevalence of intraductal hyperplasia is also observed in BPA females exposed *in utero* to BPA (0.25 mg/kg) (67). Similarly, in primates, mammary buds of female monkey offspring are denser and more developed as a result of *in utero* BPA exposure (68). In female C57/BL6 offspring, the perinatal exposure to BPA (3 μg/kg-bw) increases the number of mammary epithelial cells and TEB; and expression of progesterone receptor (PR), Wnt family member-4 (Wnt-4), and receptor activator of nuclear factor κB ligand (Rankl). These morphological and expression changes are induced by a level of exposure that approximates estimated daily BPA intake in formula-fed infants (1–13 μg/kg-bw/d) and associate with increased endpoints of breast cancer risk (69).

The exposure to bisphenols during early developmental stages imparts long-term effects. For example, a single neonatal exposure to BPA (250 μg/kg) decreases in female syngeneic BALB/c mice the number of immunoglobin IgM that recognize tumor antigens (70). BPA-exposed mice develop larger tumors with a higher proportion of regulatory T lymphocytes expressing increased levels of ERα (71). In Balb/c mice, exposure to BPA during puberty increases lateral branching and hyperplasia in adult mammary glands (72). Similarly, in female adult albino rats, BPA induces an increase in the number and size of acini and ducts in the mammary gland with hyperplasia of lining epithelial cells showing an increase in Ki-67 and caspase-3 (73). Taken together, these animal studies show that exposure to bisphenols during the perinatal, pubertal, and adult periods has the potential to alter mammary gland morphology and increase breast cancer risk.

### Human Environmental Exposures

In human biological samples that were exposed at an environmental level, BPA is detected at concentrations ranging between 0.3 and 40 nM from fetal serum and maternal plasma, respectively (74–76). Low doses of BPA exposure are defined as ≤ 5 mg/kg body weight /d (69). In addition to direct exposure from food sources, BPA crosses the placenta and is found in breast milk. Free BPA is detected in 62% of milk samples [ ≤ 0.22–10.8 ng mL(-1), median 0.68 ng mL(-1), mean 3.13 ng mL(-1)] from a group (*n* = 21) of nursing mothers (77). In a Chinese cohort, the blood BPA is higher in children (average 3.18 ± 1.66 ng/ml) compared to adults (0.2 ± 0.10 ng/ml) (78). Given their lipophilic property, bisphenols may accumulate in adipose tissue and exert long-term effects (4, 77). On the other hand, in breast adipose tissue samples provided by 36 breast cancer mastectomy patients and 14 reduction mammoplasty patients BPA concentrations are similar (0.39 vs. 0.41 ng/g, *p* = 0.74) (79).

In studies of human urine, BPA is found in 96% [1,808 adults and 868 children (2013–2014 NHANES)] of samples randomly selected. Median level of BPA in US adults is ~1.24 μg/L and higher than BPF (0.35 μg/L) and BPS (0.37 μg/L). For children, median BPA level are also higher (1.25 μg/L) than BPF and BPS (0.32 and 0.29 μg/L, respectively). Bisphenol S is found in 81% of samples representing the US and seven Asian countries (3, 9). In the 2013–2014 NHANES database, BPA is found at higher concentrations among low socioeconomic status individuals and in children. Urinary BPA concentrations of young adults (18–25 years of age) are lower than those for adults aged 26+ years, and associate with higher BMI (80).

Conjugation and deconjugation is a biochemical process that determines the levels of exposure to free and inactive bisphenols, respectively. Analysis of urine samples from a cohort of healthy full term (≥37 weeks' gestation) neonates at two intervals of age (3–6 and 7–27 days) shows that only the inactive BPA glucuronide (BPAG), but not the free BPA form, is detected with concentrations ranging from <0.1 μg/L to 11.21 μg/L (median: 0.27 μg/L). These results confirm widespread BPA exposure in healthy full-term neonates, and efficient conjugation of BPA to its readily excretable and biologically inactive BPAG as early as 3 days of age (81). However, some studies suggest that cycling of conjugation/deconjugation maintains low but sustained basal levels of free BPA in the fetus (82). Fetal hepatic conjugation is low in early but increases in late pregnancy suggesting higher risk of exposure for the fetus in the early stages of fetal development (83).

Geographical location plays a major role in determining exposure to bisphenols. In a study with Bangladeshi, first- and second-generation Bangladeshi migrants to the UK, and white British girls, the average urinary BPAG increases and is significantly higher among white British (0.007 ng/mL) and second-generation British-Bangladeshi girls (0.009 ng/mL) compared to Bangladeshi girls (0.002 ng/mL). These findings point to birthplace and growth environment as variables affecting exposure to bisphenols (84). A study within the Early Life Exposure in Mexico to Environmental Toxicants (ELEMENT) birth cohort (*n* = 120 girls, age 8–13 years), shows that BPA in the second trimester associates in offspring girls with higher peripubertal testosterone and higher odds of having a Tanner Stage >1 for breast development (OR/IQR: 2.2; 95%CI: 1.0, 4.5). The association between *in utero* BPA exposure with earlier puberty may be due childhood obesity and adiposity, two conditions prevalent in young girls of Mexican ancestry (85). Similarly, in a population of Chilean girls, the higher tertile of BPA exposure associates with higher breast density (86), a factor known to increase the risk of breast cancer.

A Chinese study, albeit with a small sample size (*n* = 50) shows that BPA is detectable in a large percentage (84%) of urine samples from adults (average 1.9 ± 1.23 ng/ml) ranging from 0.1 to 8.7 ng/ml. Concentrations of BPA in urine (creatinine-adjusted) in pregnant Chinese women receiving intravenous drip within 24 h of delivery approaches ~7.0 ng/ml compared to only 0.4 ng/ml in women who do not receive intravenous treatment (78). While these results are from relatively small groups, they raise concerns about BPA exposure in particular in pregnant women for whom BPA concentrations (~30 nM) known to stimulate proliferation of breast cells have been detected in blood. Epidemiologic studies also confirm an association between urinary BPA levels and circulating inflammation-related markers in adult populations. In elderly subjects (60 years of age or older), higher urinary BPA are positively associated with inflammation-related markers including white blood cell count, C-reactive protein (CRP), alanine (ALT) and aspartate AST) transaminase, and γ-glutamine transferase (γGTP) levels; and negatively associated with IL-10. The latter exerts anti-inflammatory and antitumor effects. These findings suggest that BPA exposure in adults induces inflammation and compromises cellular factors that protect against breast tumorigenesis (45).

### Epigenetics

#### BPA

Epigenetics refers to modifications in gene expression without changes in DNA and include changes in histone posttranslational modifications; DNA CpG methylation; and expression of non-coding RNA. Albeit limited, research data corroborate the epigenetic effects of exposure to bisphenols in breast tissue. BPA preferentially reduces CpG methylation at ERα binding genes (87). Through this DNA modification bisphenols increase accessibility of transcription factors at E2-responsive genes which contribute to breast carcinogenesis (i.e., *CCND1*). In addition, bisphenols increase expression of factors involved in epigenetic silencing of tumor suppressor genes. One such factor is enhancer of zeste homolog 2 (EZH2), which is a methyltransferase specific to histone 3 lysine 27. EZH2 expression is induced by BPA through recruitment to the EZH2 promoter of ER, mixed lineage leukemia (MLL) and CBP/P300, which contribute to transcriptional activation (88). EZH2 expression and histone H3 trimethylation are elevated in ERα-positive breast cancer cells and mammary tissue of mice exposed *in utero* to BPA (89). Increased expression of EZH2 associates with decreased nuclear expression of phospho-BRCA1 (Ser1423) and upregulation of phospho-Akt-1 (Ser473) in ~ 40% of invasive breast carcinomas. Therefore, through upregulation of EZH2, exposure to bisphenols contributes to loss of genomic stability and increased proliferation mediated respectively by loss of BRCA1 and gain of PI3K/Akt-1 activity (90). Importantly, loss of BRCA1 enhances BPA-induced ERα signaling, cell proliferation, and mammary tumorigenesis (91). These results suggest that the breast cancer response to bisphenols is magnified in women who are BRCA1 mutation carries or in subjects with epigenetic silenced *BRCA1* gene. Furthermore, DNA damage and disruption of cell cycle control by BPA associates with hypermethylation of various genes encoding factors that protect against breast cancer including TIMP metallopeptidase inhibitor 3 (*TIMP3*), which inhibits MMP involved in invasion and metastasis; checkpoint with forkhead and ring finger (*CHFR*), a tumor suppressor that delays passage into mitosis; *ESR1* encoding for ERα, whose expression is necessary to activate *BRCA1*; and immunoglobulin superfamily member 4 (*IGSF4*), which participates in cell adhesion. Overall, loss of expression of these genes through hypermethylation is an epigenetic mechanism through which bisphenols increase susceptibility to breast tumorigenesis (92). The capacity for bisphenols to predispose to breast tumorigenesis finds support in the evidence that BPA induces proliferation and hypermethylation of *BRCA1* in human non-tumor mammary epithelial cells (HMEC) (93).

Homeobox A-D genes encode for transcription factors that exert differential effects on breast cancer. HOXC6 overexpression triggers expression of tumor growth factor and associates with breast cancer. It is induced epigenetically upon exposure to BPA both *in vitro* and *in vivo*. ERα and ER-coregulators such as MLL are recruited to the HOXC6 promoter upon exposure to E2 or BPA and this triggers histone H3K4-trimethylation, histone acetylation, and recruitment of RNA polymerase II at the HOXC6 gene (94). Similarly, BPA induces expression of HOXB9, which contributes to cell proliferation. Mechanistically, ERα and the cofactors MLL-histone methylase (MLL3), CBP/P300, bind to the HOXB9 promoter at ERE in the presence of BPA leading to HOXB9 transactivation (95).

#### BPA Analogs

Bisphenol compounds exert differential DNA methylation alterations with the majority of these being ER-dependent (BPA>BPS>BPF). In particular, BPA- and BPS-induced methylome alterations associated with focal adhesion, cGMP-PKG, and cancer pathways (96). Higher proliferation in ER-positive breast cancer cells is noted following treatment with BPA or its substitute, BPS, accompanied by an ERα-dependent decrease in genomic TET-catalyzed DNA hydroxymethylation. These findings highlight the E2-like activity of BPA/BPS and the epigenetic impact on breast tumorigenesis (97). The exposure of MCF-7 cells to BPS induces DNA methylation of transposons. Upon methylation, transposon become inactive with mutation of methylated cytosines (C>T) leading to loss of transposon function (38). Additionally, BPS upregulates genes (THBS4, PPARGC1A, CREB5, COL5A3) related to breast cancer progression. The CpG methylation status of breast cancer related genes (BRCA1, CDH1, PTEN, and CCND2) is also increased (38). These results suggest that BPS exposure plays a role in the progression of breast cancer through epigenetic changes hampering DNA repair and tumor suppressor functions.

### Bisphenols and Food Components

Foods are important sources of exposure to bisphenols (Table 1) (6–9). A 24-h recall study of 1,101 girls (6–8 years of age) from the Breast Cancer and Environment Research Program (BCERP) shows increased urinary BPA levels associated with intake of grains, flour, fish, non-fresh vegetables, and poultry. Consumption of fats and oils are also positively associated with BPA exposure (98). Foods are also a vehicle of compounds that regulate the estrogenic effects of bisphenols. Unfortunately, this is an undeveloped area of research as only a few studies summarized below have examined the effects of food components in combination with bisphenols on endpoints of breast cancer.

#### Cell Culture and Xenogratfs

Genistein is the predominant isoflavone in soy. It regulates gene expression through ERα although with lower efficacy compared to E2 in ERα-positive breast cells (99). *In vitro*, genistein synergizes with bisphenols to induce estrogenic responses. In HeLa-ERα and ERβ reporter cells, the coexposure to BPA and genistein, or SF, results in increased functional and transcriptional estrogenic effects, which are abolished by ER antagonists. Genistein- and BPA-induce gene expression profiles adversely linked to breast cancer prognosis similar to those induced by E2 both at low (100 nM) and high doses (10 μM) (29). Therefore, dietary intake of genistein may enhance the estrogenic effects of bisphenols. Chronic supplementation of MCF-7 cells with genistein and BPA (50 nM) *in vitro* causes reduced expression of E2-responsive genes including MYC, EGR3, and HDAC11 associated with a decrease in H3K4me1. These changes are not reversed by removal of BPA and genistein suggesting these compounds epigenetically reprogram breast cells (100).

Resveratrol (trans-3,4,5-trihydroxystilbene; RES) is a naturally occurring phytoestrogen found in various foods including grapes and red wine. Studies related to the effects of RES in breast cells under conditions of exposure to BPA are limited. However, in E2-responsive MCF-7 CV cells, RES reverses cell proliferation induced by E2 or BPA by down-regulating the expressions of ERα, IGF-1R, p-IRS-1, and p-Akt1/2/3, and cyclin D1 at both transcriptional and translational levels (101). Whether or not RES is effective in breast tissue under conditions of exposure to bisphenols warrants further investigation.

Curcumin is a curcuminoid component of turmeric which has been extensively studied in cancer prevention and treatment. Curcumin inhibits the proliferative effects of BPA on MCF-7 cells. The BPA-induced upregulation of oncogenic miR-19a and miR-19b, and the dysregulated expression of miR-19-related downstream proteins, including PTEN, p-AKT, p-MDM2, p53, and proliferating cell nuclear antigen are reversed by curcumin. These results suggest that curcumin modulates miR-19/PTEN/AKT/p53 axis to exhibit its protective effects against BPA-associated breast cancer promotion (102).

The compound 3,3'-diindolylmethane (DIM) originates from condensation of two indole-3-carbynol (I3C) moieties in the acidic environment of the stomach. Foods rich in I3C include broccoli, cabbage and cauliflower. The co-treatment with DIM (20 μM) prevents E2- and BPA-induced cell proliferation, EMT, migration, and invasion of MCF-7 cells. Moreover, DIM decrease CXCR4 protein expression. These *in vitro* effects of DIM are also seen in a xenograft mouse model transplanted with MCF-7 breast cancer cells (103). Another compound found in cruciferous vegetables is the flavanol kaempferol. Whereas, BPA (0.1–10 μM) and E2 (0.01–0.0001 μM) induce cell proliferation of VM7Luc4E2 cells, these responses are antagonized by co-treatment with kaempferol (30 μM) or DIM (15 μM). BPA inhibits ROS production and apoptosis of VM7Luc4E2 cells similar to E2, but the co-treatment with kaempferol or DIM increases ROS production and apoptosis (104). These results suggest DIM and kaempferol may be an effective cruciferous components for the prevention of metastatic breast cancer resulting from exposure to bisphenols.

Naringenin is a flavanone found in grapefruit and oregano. It binds to ERα and hampers cell proliferation by activating caspase-3. Also, the BPA-induced AKT activation is antagonized by naringenin, which prevents the antiapoptotic effects of BPA (105). Mechanistically, naringenin induces ERα protein accumulation by preventing proteasomal receptor degradation via activation of p38/MAPK pathway (106).

#### Animal Models

Animal studies suggest that prepubertal exposure to genistein exerts preventative effects against endpoint associated with breast tumorigenesis. Results of a rodent study with lactating dams (Sprague-Dawley rats) shows that dietary genistein (250 ppm/diet) to achieve physiologically relevant serum concentrations (~700 pmol/L) induces in offspring expression of factors involved in cancer prevention including MMP3; rho-associated coiled-coil containing protein kinase 2 (ROCK2); neurosecretory protein VGF 8a (VGF), serine (or cysteine) proteinase inhibitor clade A (SERPINA1); ubiquitin carboxyl-terminal hydrolase L5 (UCH1); SET domain containing 2 (SETD2); and protein tyrosine phosphatase receptor type K (PTPRK) (107). The maternal nutrient supplementation with genistein (250 ppm) counteracts BPA-induced DNA hypomethylation in early development (108). In prepubertal rats, the expression of annexin A2, VEGFR and Akt1 are increased in mammary tissue following treatment with BPA. Conversely, the dietary treatment with genistein exerts repressive effects on expression of these factors which contribute to various cancer processes such as angiogenesis, proliferation, and metastasis. These differential effects of BPA and genistein are proposed to contribute at least in part to their opposing effects on mammary carcinogenesis although both BPA and genistein elicit estrogenic effects (109). Two genes associated with improved survival, *HPSE* and *RPS9*, are hypomethylated in mammary tissue of rats exposed in prepuberty respectively to genistein alone or in combination with BPA (110).

A diet high in saturated fat (HFD) is negatively associated with breast cancer survival (111). During gestation, feeding a HFD along with a low exposure to BPA (25 μg/kg BW/day) increases mammary tumor incidence in offspring, while reducing tumor-free survival time compared with the HFD alone. These *in utero* procarcinogenic effects of BPA associate with epigenetic reprogramming via CpG hypomethylation of Kcnv2 and hypermethylation of Car7 in mammary tissue of female offspring. These data suggest that concurrent exposure to a HFD and BPA during pregnancy increases mammary tumor incidence in offspring associated with epigenetic dysregulation (112).

#### Clinical Studies

Switching from a diet of canned foods or foods packaged in plastic to a diet of fresh foods reduces exposure to bisphenols. In a food intervention study, the urine levels of BPA metabolites decreased by ~65% during the fresh foods intervention within 3 days. From this study, it seems that restricting intake of packaged and canned food is an effective approach to reducing exposure to bisphenols (113).

The Genes, Environment, and Health Initiative is a longitudinal study of girls enrolled at 6–7 years of age and followed through puberty. From the high-BPA group (average urinary BPA = 17.5 ± 11.2 ng/g- creatinine adjusted), several factors associated with cancer are increased and include ankyrin 2, a cytoskeletal protein involved in metastasis and migration; antigen Ki-67, which participates in cell proliferation; and E3 ubiquitin-ligase, talin 2, transient receptor potential channel 5 (TRPC5), mitogen-activated kinase kinase 4 (MKK4), and zinc finger 185 which are involved in cancer development. In blood of the high-genistein girls (average 1.3 μg/g -creatinine adjusted), the nucleolar 7 and PR domain zinc finger 5 (PRDM5) factors with anticancer roles are upregulated. Differential gene regulation in girls with high concentrations of BPA and genistein are consistent with reported roles of BPA and genistein respectively in mammary cancer promotion and prevention. In blood of girls with high genistein concentrations in their urine, two proteins associated with cancer were down regulated: endothelin-converting enzyme (ECE-1) and eukaryotic translation initiation factor 3 subunit J (EIF-3). Conversely, nucleolar 7 and PR domain zinc finger 5 (PRDM5) are proteins that are upregulated in high-genistein girls. The nucleolar 7 gene is a tumor suppressor gene antagonizing the angiogenic process. PRDM5 has growth suppressive activities and is silenced in breast, ovarian, liver, lung, colon, and other cancers. All four proteins should be considered as biomarkers of susceptibility for genistein/soy and cancer prevention (114).

## Discussion and Conclusions

**Figure 2:**
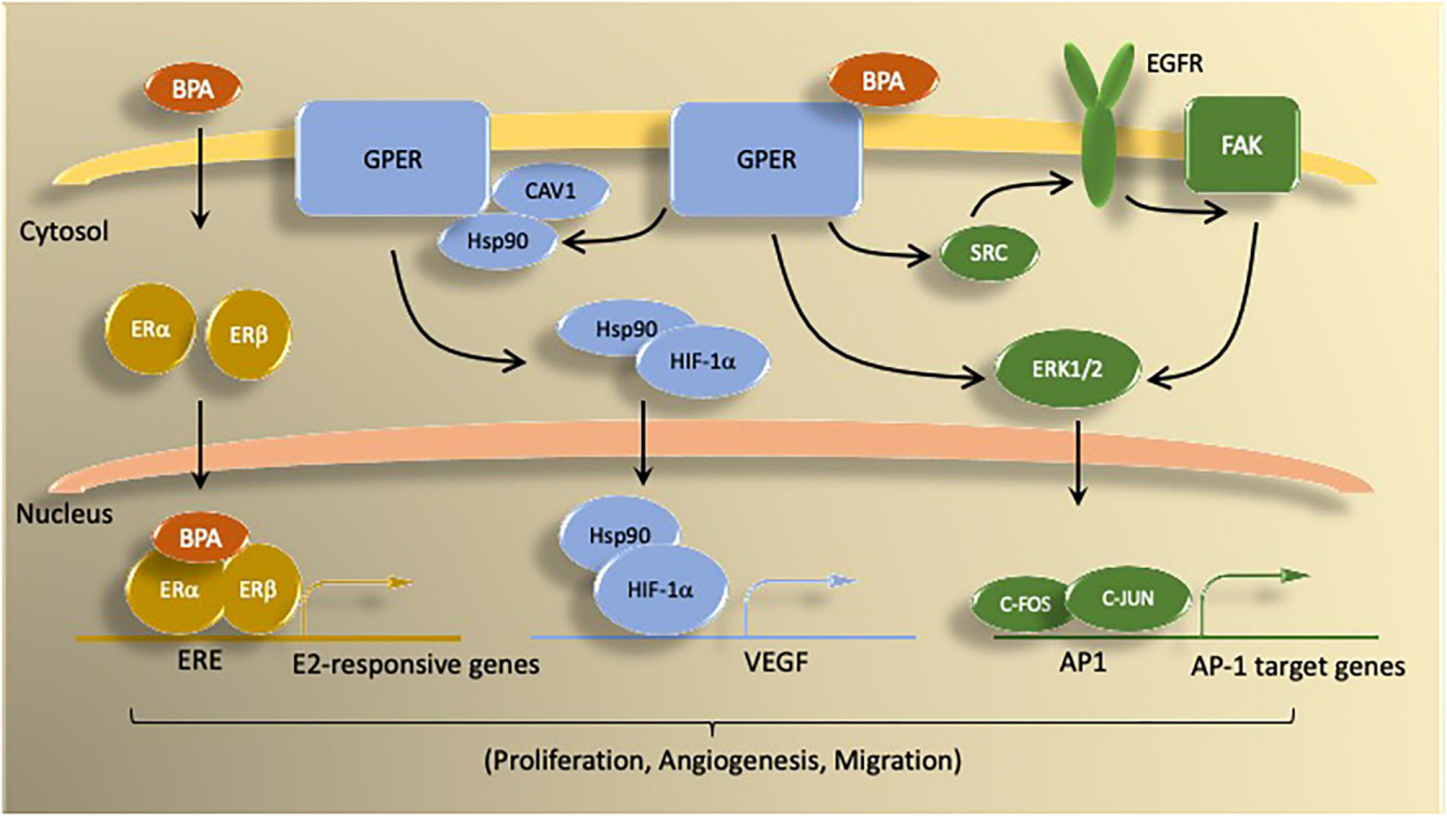
Summary of BPA mechanisms of action *via* estrogen receptors. BPA activates both genomic and non-genomic estrogen signaling pathways. Genomic signaling involves activation of the nuclear ER proteins ERα and ERβ. Nuclear ER bind as either homo- or heterodimers to induce transcription of genes controlled by estrogen responsive elements (ERE). Non-genomic actions of BPA involve signaling through G protein coupled estrogen receptor (GPER) molecules, which activate signal transduction pathways (e.g., ERK1/2) *via* kinase activity. BPA binding to GPER activates downstream signaling by epidermal growth factor receptor (EGFR) and focal adhesion kinase (FAK). GPER signaling through ERK1/2 stimulates transcription of c-FOS-dependent genes. Under hypoxic conditions, BPA activation of GPER promotes HIF1α-dependent induction of *VEGF*, which is associated with increased proliferation, migration, and angiogenesis.

Bisphenols exhibit vast actions on endocrine regulation (Figure 2) and appear to contribute to the progression of ER positive and ER-negative breast tumors. However, several questions have not been conclusively addressed in previous studies. Given the potential estrogenic effects of BPA in neonates, chemical analogs such as BPF, BPAF, and BPS have been introduced in industrial processes. However, in available, albeit limited, studies these analogs demonstrate similar or even stronger estrogenic, and possibly carcinogenic, effects as the parent BPA compound. Therefore, research that addresses the effects of dose and timing of exposure to bisphenol analogs is warranted. This is particularly important as exposure to bisphenols appears to impact epigenetic gene expression by upregulating genes involved in cancer processes while silencing genes with tumor suppressor functions (i.e., BRCA1). Of particular concern is the fact that removal of bisphenols may not be sufficient to reverse the epigenetic marks on genes. Therefore, the potential exists for exposure to bisphenols at different stage of life (i.e., gestation, prepuberty, adult) to permanently silence genes with tumor suppressor functions, while eliciting the constitutive activation of oncogenes.

Another important area for investigation pertains to the effects of bisphenols on breast cancer subtypes. While most emphasis is placed on the estrogenic effects of bisphenols through activation of ER, it appears bisphenols can also promote growth of ER-negative and TNBC cells through non-ER mediated mechanisms. Under the assumption that the timing and dose of bisphenol exposure in animal models mimics that of humans, one of the most vulnerable populations is infants, who regardless of their nutrition source (placenta *in utero*, then breastmilk or formula) may be exposed to bisphenols while simultaneously undergoing the most significant period of development. Further, the results summarized here indicate that individuals carriers of mutations or epigenetic marks (i.e., CpG methylation) in breast cancer genes (i.e., BRCA1) may be at higher risk from exposure to bisphenols.

Finally, more emphasis should be placed on research about foods (and their packaging) as vehicles of exposure to bisphenols and bioactive components that prevent the biochemical changes induced in breast tissue by bisphenols. To date, research about the effects of diet and bioactive food components on bisphenol-related breast tumorigenesis is scarce making it difficult to make any clinically relevant conclusions about surveillance and dietary interventions. However, a few studies have highlighted the possible preventative effects of compounds found in cruciferous vegetables, grapefruit, grapes, and turmeric. While some cell culture studies have raised concerns about the additive effects of soy genistein with bisphenols, most animal studies suggest it exerts a preventative effect when exposure occurs during the prepubertal phase of life. Ultimately, as clinicians wait for further research, and bisphenols remain ubiquitous in the environment, it is advisable to limit exposure to BPA by avoiding heating food in plastic containers and avoiding the use of canned food and foods packaged with polycarbonate plastics for breast cancer risk reduction.

## Author Contributions

BS, AB, DR, LN, MD, and OS contributed to the conception and development of the manuscript. BS, AB, and DR had primary responsibility for the writing of the manuscript. LN was responsible for the clinical content of the work and contributed to the writing and review of the manuscript. All authors contributed to the article and approved the submitted version.

## Conflict of Interest

The authors declare that the research was conducted in the absence of any commercial or financial relationships that could be construed as a potential conflict of interest.
